# Computer-Aided Diagnosis of Equine Temporomandibular Joint Osteoarthritis Using Machine Learning Integrating Computed Tomography Findings and Synovial Fluid Biomarkers

**DOI:** 10.3390/ani16060932

**Published:** 2026-03-16

**Authors:** Tomasz Jasiński, Marta Borowska, Edyta Juszczuk-Kubiak, Bernard Turek, Michał Kaczorowski, Mateusz Bąk, Julia Żuk, Małgorzata Domino

**Affiliations:** 1 Department of Large Animal Diseases and Clinic, Institute of Veterinary Medicine, Warsaw University of Life Sciences, 02-787 Warsaw, Poland; tomasz_jasinski@sggw.edu.pl (T.J.); bernard_turek@sggw.edu.pl (B.T.); 2 Institute of Biomedical Engineering, Faculty of Mechanical Engineering, Białystok University of Technology, 15-351 Bialystok, Poland; julia.zuk@sd.pb.edu.pl; 3 Department of Biotechnology, Prof. Wacław Dąbrowski Institute of Agricultural and Food Biotechnology-State Research Institute, 02-532 Warsaw, Poland; edyta.juszczuk-kubiak@ibprs.pl (E.J.-K.); mateusz.bak@ibprs.pl (M.B.); 4 Private Equine Practice, 05-825 Grodzisk Mazowiecki, Poland; mskaczorowski@gmail.com

**Keywords:** temporomandibular diseases, TMJ, OA, classification, horse

## Abstract

Horses presenting with temporomandibular joint (TMJ) dysfunctions are often clinically evaluated for TMJ osteoarthritis (OA). Due to the unique characteristic of TMJ-related pain, the clinical diagnosis of equine TMJ OA is challenging; however, it may be supported by computer-aided tools incorporating biomarker data. This study aims to evaluate a machine learning-based approach to address a binary classification distinguishing healthy TMJs from TMJ OA. Among 50 equine cadaver heads, 82 TMJs were included and annotated as healthy or OA based on histological and computed tomography (CT) findings. For each TMJ, nine CT findings were assessed, and synovial fluid was collected for the evaluation of twelve biomarkers. Using a biomarker dataset, correlations among biomarkers were calculated and supported with a mixed-effects logistic regression model. Using a combined dataset, twelve machine learning models, incorporating two feature selection methods and six classification algorithms, were evaluated. Specific biomarker levels showed predominately positive correlations with TMJ OA, age, and with each other; however, only age had a significant effect on OA assignment in the mixed model. The best-performing machine learning model achieved an accuracy of 0.82 and an area under the curve (AUC) of 0.85 for binary TMJ classification. The proposed classification model outperforms conventional diagnostic methods and may therefore be considered beneficial in aiding the diagnosis of equine TMJ OA.

## Simple Summary

Osteoarthritis (OA) is a painful, degenerative joint disease that affects temporomandibular joints (TMJs). In horses, however, clinically linking pain in the head region to TMJ dysfunction is often challenging. This diagnosis may be supported by computer-aided tools incorporating biomarker data. The study aims to introduce a machine learning-based approach to support the distinction between healthy TMJs and those affected by OA. To achieve this aim, a dataset was created by combining nine computed tomography (CT) findings with twelve synovial fluid biomarkers collected from 82 TMJs. Each TMJ was annotated as healthy or as having TMJ OA based on histological changes co-occurring with CT findings. Using a biomarker dataset, correlations among biomarkers were calculated and supported with a mixed-effects logistic regression model. Using a combined dataset, twelve machine learning models were evaluated, incorporating two feature selection methods and six classification algorithms. Specific biomarker levels showed predominately positive correlations with TMJ OA, age, and with each other; however, only age had a significant effect on OA assignment in the mixed model. The best-performing model achieved an accuracy of 0.82 and an area under the curve of 0.85 for distinguishing between healthy TMJs and TMJ OA. This classification model achieved better performance than conventional TMJ OA diagnosis based only on CT findings.

## 1. Introduction

Temporomandibular diseases (TMDs) are rarely reported in horses [1]; however, when described in the literature, they predominately include septic temporomandibular joint (TMJ) arthritis [2,3,4,5,6,7,8,9,10,11,12,13] and primary TMJ osteoarthritis (OA) [13,14,15,16,17,18,19,20,21,22]. Whether the infrequent reporting of TMDs is attributable to a truly low prevalence or to misdiagnosis [22], horses presenting with masticatory problems, head-shaking syndrome, or poor performance are clinically evaluated for TMJ dysfunctions [1].

Horses with septic TMJ arthritis are typically presented to equine veterinarians because of head wounds and masticatory problems [1]. These include moderate TMJ dysfunctions—such as difficulties eating [3,13], reduced appetite [9], or quidding [12]—as well as severe TMJ dysfunctions, including inability to open the mouth [10] or masticate [2], and inappetence [4,10]. Conversely, horses with primary TMJ OA are usually clinically evaluated due to joint swelling and mild to moderate TMJ dysfunctions [1]. These signs are more often associated with performance-related issues—such as head-shaking syndrome [17,21], problems with bit contact when riding [15,16], and behavioral changes [16,17]—than with overt masticatory problems, including difficulties eating [13], quidding [14,17], or clunking/clicking sounds when eating [16,17]. These clinical signs prompt equine veterinarians to perform a complete physical and oral examination [23], as many clinical signs potentially related to TMDs may also be attributable to dental disease [24,25] or to other more common orthopedic, cardiovascular, respiratory, and neurological conditions [23,24]. Consequently, horses present a unique challenge in the determination of TMJ-related pain and diseases [24], since attributing discomfort to the TMJ is largely a diagnosis of exclusion [23].

In equine TMD diagnostic protocols, intra-articular analgesia, although considered necessary [23], is rarely reported as a diagnostic test to localize the clinical signs of dysfunction [1]. It may be performed when evident TMJ dysfunction signs are detectable [15,16,26], but may be refrained from when clinical signs are absent at the time of examination [17]. As a result, intra-articular analgesia may yield equivocal clinical findings in some cases [24]. The lack of objective assessment of TMJ dysfunction may also stem from the unique characteristics of the equine TMJ. Horses may mitigate TMJ-related signs of inflammation by altering their eating behavior and preferentially masticating on the contralateral side [27]. Moreover, they appear less susceptible to overt manifestations of TMJ-related pain than to pain arising from peripheral joints [21]. Additionally, although horses with TMJ inflammation may avoid pressure from the bit on the affected side, riders may partially compensate for instability or bit-contact problems by increasing rein tension on the unaffected side [28]. For these reasons, Carmalt et al. [24] suggested that the introduction of a readily available biomarker would be beneficial in aiding the diagnosis of equine TMDs.

This strategy has been extensively investigated in human medicine, involving both conventional studies in which various proteins have been evaluated to categorize, quantify, and prognosticate TMJ inflammation [29,30], as well as more recent research on computer-aided diagnosis of TMJ OA [31,32]. This approach assumes that different TMDs alter synovial fluid composition, including changes in cytokine, chemokine, growth factor, enzyme, and other mediator levels, and that these changes offer opportunities to improve diagnostic accuracy and monitor response to treatment [29]. In the first context, qualitative and quantitative differences in synovial fluid composition between healthy individuals and those with TMJ OA have been widely described in humans [33,34,35,36,37,38,39,40,41,42,43,44,45,46]. In the second context, complex TMD patterns—those that elude traditional statistical approaches—have been investigated using machine learning algorithms applied to integrated datasets comprising heterogeneous radiographic findings and synovial fluid biomarkers [31,32]. In these studies, radiographic findings quantified from computed tomography (CT) images of the TMJ were combined with biomarker levels to evaluate the performance of machine learning models for computer-aided diagnosis of human TMJ OA [31,32]. The authors highlighted that comprehensive integration of artificial intelligence tools with heterogeneous biological data acquisition, extraction, and processing may allow accurate prediction of patient-specific TMJ OA status [31].

Among specific TMJ OA biomarkers, elevated levels of monocyte-macrophage-derived cytokines—such as interleukins (ILs) including IL-1β [33,34,35,36], IL-6 [33,34,35,37], IL-8 [33,34], IL-12 [35,36], IL-17 [35,37], and tumor necrosis factor α (TNF-α) [33,34,35,36]—have been reported in the synovial fluid of human patients with TMJ OA. These pro-inflammatory cytokines can stimulate the expression of degradative enzymes and inflammatory mediators, leading to TMJ inflammation and degradation of articular cartilage and subchondral bone [47]. Moreover, IL-1β upregulates chemokine levels—particularly monocyte chemoattractant protein 1 (MCP-1), which promotes recruitment of monocytes to the TMJ synovium and thereby increases the number of cytokine-producing cells [38]. Notably, the production of these inflammatory mediators is induced by transforming growth factor β1 (TGF-β1), whose signaling pathway plays a critical role in OA development and progression by driving chondrocytes toward hypertrophy, promoting subchondral bone remodeling, and stimulating synovial cell expansion and fibrosis [39,48]. In addition, TGF-β1 upregulates the production of vascular endothelial growth factor (VEGF), resulting in increased angiogenesis [40,48] and matrix metalloproteinase (MMP) activity, whose evaluated activity contributes to extracellular matrix (ECM) degradation in articular cartilage [41,42]. MMPs—particularly MMP-3 and MMP-13—are proteolytic enzymes responsible for the degradation of ECM components and are implicated in TMJ inflammation and pain modulation [42,43,44]. Furthermore, increased levels of prostaglandin E_2_ (PGE_2_) in synovial fluid have been reported in chronic OA and are associated with TMJ pain and masticatory dysfunction [45], making PGE_2_ an important biomarker in studies of TMJ-related pain [46].

It can be observed that among the synovial fluid biomarkers identified in human TMJ OA, only IL-1 [24], IL-6 [24,49], IL-8 [24], TNF-α [24,49], TGF-β [24,49], and PGE_2_ [22] have been investigated in equine TMJs, leaving a substantial gap for further research [50]. Notably, levels of IL-1, IL-6, IL-8, TNF-α, and TGF-β were measured in the synovial fluid of horses with no history or clinical signs of TMDs to determine whether age or dental disease influences cytokine levels in clinically normal TMJs [24]. Subsequently, levels of IL-6, TNF-α, and TGF-β were assessed in the synovial fluid of clinically normal horses with experimentally induced acute inflammation to investigate TMJ inflammatory responses compared with those of an appendicular joint, represented by the metacarpophalangeal joint [49]. Afterwards, the PGE_2_ level was measured in the synovial fluid of clinically healthy horses with experimentally induced TMJ overload to evaluate whether dental procedures should be limited to a duration shorter than one hour to avoid TMJ damage [22]. However, none of the existing studies on synovial fluid biomarkers in equine TMJs has considered radiographic imaging of TMJs, and the application of biomarkers in computer-aided diagnosis of equine TMDs remains underdeveloped.

Therefore, this study combines CT findings of equine TMJs with biomarker levels in TMJ synovial fluid, aiming to evaluate a machine learning-based approach for addressing a binary classification problem: distinguishing healthy TMJs from those affected by OA.

## 2. Materials and Methods

### 2.1. Study Design

A prospective cross-sectional study was conducted on 50 equine cadaver heads collected post mortem between May and June 2023 at a commercial slaughterhouse in Poland. The heads were transported to the Equine Clinic at the Warsaw University of Life Sciences, where TMJ palpation, CT examination, and tissue sample collection were performed to qualify the joints for research.

The inclusion criteria were macroscopically intact joint capsules, complete CT scans of the TMJs, successful synovial fluid collection without blood contamination, and complete histopathological slides without signs of autolysis. The exclusion criteria were wounds in the TMJ or orbital area, disruption of the joint capsule, fractures of the coronoid process, mandibular condyle, or temporal bone, TMJ luxation, dentigerous cysts in the TMJ area, radiological signs of TMJ neoplasia, missing synovial fluid samples, and missing or poor-quality histological tissue samples. Based on these eligibility criteria, 9 heads were excluded due to a wound in the orbital area penetrating the TMJ (*n* = 2), disruption of the joint capsule and lack of synovial fluid collection (*n* = 4), fracture of the mandibular condyle (*n* = 1), as well as missing histological slides and/or blood contamination in synovial fluid (*n* = 2). Consequently, 41 heads (82 TMJs) from warmblood horses (23 females and 18 males), aged 1 to 25 years (mean ± standard deviation (SD): 10.2 ± 7.6 years), met the inclusion criteria and were used to create a dataset for machine learning model development.

**Figure 1:**
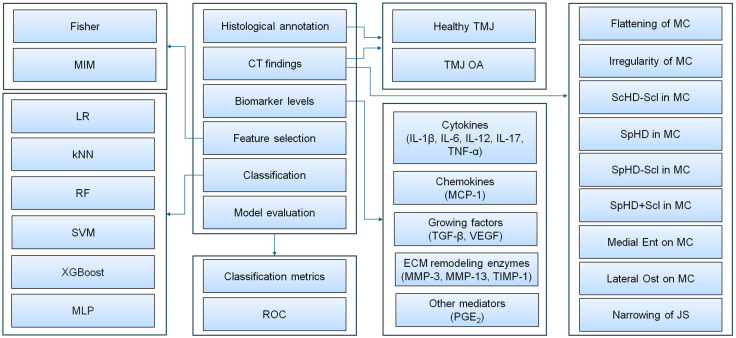
Visualization of the workflow used for computer-aided diagnosis of equine temporomandibular joint osteoarthritis (TMJ OA). Footnotes: CT—computed tomography; Ent—enthesiophytes; ECM—extracellular matrix; IL—interleukin; JS—joint space; kNNs—k-nearest neighbors; LR—logistic regression; MC—mandibular condyle; MCP-1—monocyte chemoattractant protein 1; MIM—mutual information maximization; MLP—multilayer perceptron; MMP—matrix metalloproteinase; Ost—osteophytes; PGE_2_—prostaglandin E_2_; RF—random forest; ROC—receiver operating characteristic; ScHD—scattered regions of hypodensity; Scl—sclerosis (surrounding hyperdensity); SphHD—spherical region of hypodensity; SVM—support vector machines; TGF-β—transforming growth factor β1; TIMP-1—tissue inhibitor of metalloproteinases 1; TNF-α—tumor necrosis factor α; VEGF—vascular endothelial growth factor; XGBoost—extreme gradient boosting; −—without; +—with.

Each record, representing a single TMJ, was annotated for the presence (1) or absence (0) of TMJ OA using a histological grading scale [51] and for the presence (1) or absence (0) of specific CT findings [51]. Detailed histopathological and CT results for these TMJs, which contributed directly to label definition, have been reported in a related study by our research group [51]. In the present study, the histopathological classification and CT data were reused, while the biomarker data are reported here for the first time. As a result, 41 TMJs were annotated as healthy (control group) and 41 TMJs as affected by OA (comparison group). The equal balance between healthy TMJs and those affected by OA did not result from the selection criteria. The control group included histopathologically confirmed OA-free TMJs, characterized by the absence of inflammation features of the joint capsule, regardless of other histopathological findings and CT findings. The comparison group included histopathologically confirmed TMJ OA, characterized by the presence of inflammation features of the joint capsule in combination with other histopathological findings and CT findings. Each record was characterized by 9 CT findings and 12 biomarker levels, resulting in a dataset of 21 features with 82 realizations. A machine learning-based approach was applied to this combined dataset for binary classification (healthy TMJ vs. TMJ OA). Within this framework, two feature selection methods and six machine learning algorithms were assessed. The performance of each classification model was evaluated using standard classification metrics and receiver operating characteristic (ROC) analysis. The workflow of equine TMJ OA classification used in this study is schematically presented in Figure 1.

### 2.2. CT Image Acquisition and Assessment

CT examination was performed using a 64-row Revolution CT scanner (GE Healthcare, Chicago, IL, USA) with the following acquisition parameters: helical scan mode, current of 275 mA; voltage range 70–140 kV using GSI-QC mode; gantry rotation of 0.08/s/HE+; table travel of 39.4 mm/rotation; pitch of 0.984:1; slice thickness of 2.5 mm; and slice thickness after reconstruction of 0.625 mm. The reconstruction field of view (FOV) was 370 mm, and the matrix size was 512 × 512. The scan length was adjusted to match the horse’s head length; consequently, the number of CT images was also tailored accordingly. The CT images were stored in DICOM format.

**Figure 2:**
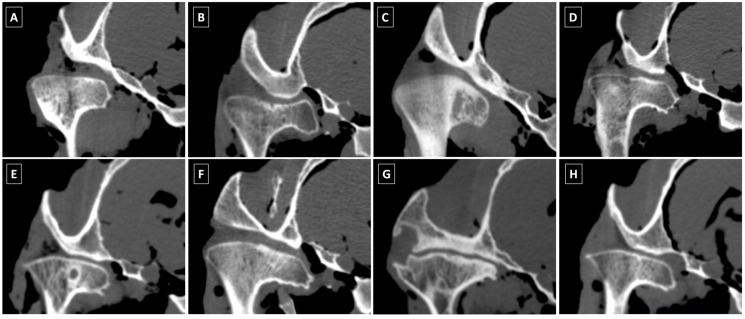
Illustration of computed tomography (CT) findings assessed in equine temporomandibular joints (TMJs) representing (**A**) flattening of the mandibular condyle (MC); (**B**) irregularity of MC; (**C**) scattered regions of hypodensity in MC without sclerosis (ScHD − Scl in MC); (**D**) spherical regions of hypodensity in MC without sclerosis (SphHD − Scl in MC); (**E**) spherical regions of hypodensity in MC with sclerosis (SphHD + Scl in MC); (**F**) enthesiophytes on the medial aspect of MC (Medial Ent on MC); (**G**) osteophytes on the lateral aspect of MC (Lateral Ost on MC); and (**H**) narrowing of the joint space (JS).

The CT images were reviewed in bone (window ±1500, level 300) and soft tissue (window ±300, level 50) windows using multiplanar reconstruction in Osirix MD software, version 12.0 (Pixmeo SARL, Bernex, Switzerland), following a CT findings extraction template described in a related study from our research group [51]. The presence (1) or absence (0) of 9 specific CT findings was annotated for each joint. Flattening [16,20] and irregularity [13,16] referred to alterations in the shape and contour of the subchondral bone in the mandibular condyle (MC), respectively. Flattening of MC was considered a potential ‘CT anatomical variations’ [16]. Scattered regions of hypodensity in MC (ScHD − Scl in MC) were noted without sclerosis (surrounding hyperdensity) and recorded as a potential ‘CT anatomical variations’ [16]. Subchondral bone cysts, represented by spherical regions of hypodensity in MC (SphHD in MC) [13,15,16,19], were annotated separately, regardless of the presence or absence of sclerosis, and categorized into two groups: those with surrounding sclerosis (SphHD + Scl in MC) and those without (SphHD − Scl in MC) [16]. New bone formations in the form of enthesiophytes (Medial Ent on MC) or osteophytes (Lateral Ost on MC) were identified on the medial and lateral aspects of MC, respectively, with Medial Ent on MC considered a potential ‘CT anatomical variations’ [16]. Narrowing of the joint space (JS) was defined as a decreased distance between MC and the zygomatic process of the temporal bone [13]. The CT findings considered in the evaluated classification models are summarized in Table 1 and illustrated in Figure 2.

### 2.3. Synovial Fluid Collection and Analysis

TMJ arthrocentesis was performed using a blind technique, as used by Norvall et al. [52]. A 20-gauge, 40 mm needle was inserted into the dorsal compartment of the TMJ, and the synovial fluid was aspirated into a dry syringe. In all samples, synovial fluid was clear and straw-yellow. Samples were collected in dry Eppendorf type tubes (Sarstedt AG & Co. KG, Nümbrecht, Germany) and stored at −80 °C until the assays were performed.

Levels of cytokines (IL-1β, IL-6, IL-12, IL-17, and TNF-α), chemokine (MCP-1), growth factors (TGF-β and VEGF), enzymes (MMP-3, MMP-13, and tissue inhibitor of metalloproteinase 1 (TIMP-1)), and other mediators (PGE_2_) were determined using equine-specific enzyme-linked immunosorbent assay (ELISA) kits (ELK Biotechnology CO. Ltd., Sugar Land, TX, USA). Assay catalog numbers, sensitivities, detection ranges, intra-assay coefficients of variation (CVs), and inter-assay CVs are summarized in Table 2. Biomarker levels below the detection limit were set to 0 for statistical analysis and further modeling.

All reagents, standards, and samples were prepared according to the manufacturer’s instructions and equilibrated to room temperature prior to use. The required number of microplate strips was assembled, and unused strips were stored at 2–8 °C. Samples were analyzed in duplicate on the same microplate to minimize inter-assay variability, in accordance with a within-subject study design. Standards and samples were added to the appropriate wells, followed by the addition of biotinylated primary antibody and streptavidin-horseradish peroxidase except in blank control wells. Plates were incubated for 60 min at 37 °C and then washed five times with wash buffer using an automated BioTek 405 TS microplate washer (Agilent Technologies Inc., Santa Clara, CA, USA). Substrate solutions A and B were then added, and the plates were incubated for 10 min at 37 °C in the dark. The addition of stop solution terminated the reaction, and absorbance was measured immediately at 450 nm using a BioTek Synergy H1 microplate reader (Agilent Technologies Inc., Santa Clara, CA, USA). Levels of IL-1β, IL-6, IL-12, IL-17, TNF-α, MCP-1, TGF-β, VEGF, MMP-3, and PGE_2_ were expressed in pg/mL, whereas levels of MMP-13 and TIMP-1 were expressed in ng/mL.

### 2.4. Statistical Analysis

Data series were created by grouping CT findings and biomarker levels into healthy TMJ and TMJ OA groups based on the absence (0) or presence (1) of TMJ OA findings, as determined by a histological grading scale co-occurring with at least one CT finding [51]. For each feature, data distribution was assessed independently using the Shapiro-Wilk test. Because not all data series followed a normal distribution, results are presented as medians and ranges (minimum-maximum values). CT findings and biomarker levels were compared between healthy TMJ and TMJ OA groups. When both data series were normally distributed, an unpaired *t*-test with Welch’s correction was applied. When one or both data series did not follow a normal distribution, the Mann-Whitney test was used. Differences were considered statistically significant at *p* < 0.05.

Spearman’s rank correlation coefficient (ρ) was calculated to assess the associations between TMJ OA annotations (0, 1), horses’ age, sample laterality (left TMJ coded as 0 and right TMJ coded as 1), and biomarker levels. Correlations were considered significant for *p* < 0.05. Statistical analysis was performed using GraphPad Prism version 6 (GraphPad Software Inc., San Diego, CA, USA).

### 2.5. Mixed-Effects Logistic Regression Model Application

A mixed-effects logistic regression model was applied to the biomarker dataset (12 biomarker levels with 82 observations), combined with TMJ OA annotations (0, 1), horses’ age, and sample laterality (left TMJ coded as 0 and right TMJ coded as 1). The model was applied using a generalized linear mixed model (GLMM) with a logit link function and a random intercept for horses. The multivariate GLMM estimated the probability of TMJ OA (coded 0/1) as a function of age, laterality, and biomarker levels according to the following formula (1):

(1) $$TMJ\;{OA}_{ij}\sim Bernoulli\left(p_{ij}\right),\;logit\left(p_{ij}\right)=\beta_{0}+u_{j}+\beta_{Age}{Age}_{ij}+\beta_{Lat}{Laterality}_{ij}+\sum_{k=1}^{12}\beta_{k}{Biomarkers}_{k,ij}$$

where $u_{j}\sim N(0,\sigma_{horse}^{2})$ represents the random intercept for horse (horse ID Code), modeling within horse correlation. The random effects variance component ($\sigma_{horse}^{2}$) quantified between-horse variability and enabled proper adjustment for the paired structure of the data. All biomarkers were z-standardized (mean-centered and scaled to one standard deviation) prior to modeling, so fixed-effect coefficients and corresponding odds ratios (ORs) represent changes per 1 standard deviation increase in each biomarker. Age was treated as a continuous predictor (years), and laterality as a binary indicator (left = 0, right = 1).

The model was implemented within Python version 3.11, using the Bayesian Model Building Interface (Bambi) version 0.17, which provides a high level interface to PyMC version 5.28 for full Bayesian inference. Sampling from posterior distributions was performed using the No-U-Turn Sampler (NUTS), an adaptive Hamiltonian Monte Carlo algorithm. Four Markov chains were run in parallel, each with 3000 warm up (tuning) iterations and 2000 posterior draws, with a target acceptance rate of 0.97 to ensure stable exploration of the posterior. Convergence was assessed using standard diagnostics implemented in ArviZ version 0.8, including the Gelman-Rubin statistic (R hat ≈ 1.00 for all parameters), effective sample size, and energy diagnostics. No divergences were recorded.

Associations between TMJ OA annotations, horses’ age, sample laterality, and biomarker levels were expressed as OR with 95% highest density intervals (HDIs) for interpretability and corresponding credibility indicators (true, false). HDIs were chosen instead of classical confidence intervals (CIs) because Bayesian models infer parameters as probability distributions rather than fixed quantities. OR values were considered credible when they did not include 1.0 (lower bound of the HDI > 1.0; higher bound of the HDI < 1.0).

### 2.6. Machine Learning Models Application

Classification was performed independently using six classifiers: logistic regression (LR), k-nearest neighbors (kNNs), random forest (RF), support vector machines (SVMs) with radial basis function kernels, extreme gradient boosting (XGBoost), and multilayer perceptron (MLP) neural networks. The models were trained using the standard parameter settings implemented in the scikit-learn library version 1.4.2 (https://scikit-learn.org/stable/, accessed on 6 March 2026) within Python version 3.11.

All algorithms employed binary classification (healthy TMJ vs. TMJ OA) based on the absence (0) or presence (1) of TMJ OA findings, as determined by a histological grading scale co-occurring with at least one CT finding [51]. Since two TMJs originate from the same horse, the data are not fully independent; therefore, the Stratified Group K-fold (StratifiedGroupKFold() function from the scikit-learn library version 1.4.2 (https://scikit-learn.org/stable/, accessed on 6 March 2026) setting was used when dividing data into folders to group data by horse and thus prevent data leakage between folds, ensuring that features from the same horse (e.g., left and right TMJs) were not included in both the training and testing folds. To further prevent data leakage, all preprocessing steps—including z-standardization and filter-based feature selection—were performed strictly within each training fold using a scikit-learn pipeline.

Feature scaling was applied within the preprocessing pipeline to standardize the data using the StandardScaler() function from the scikit-learn library version 1.4.2 (https://scikit-learn.org/stable/, accessed on 6 March 2026) within Python version 3.11. Feature selection within each fold rather than globally was performed on the training fold, not on the entire dataset, prior to classification. Two feature selection methods—the Fisher method and the mutual information maximization (MIM) method—were applied to the created dataset (21 combined CT and biomarker features with each training fold). The top 30% of the most important features were determined independently for each method. Feature selection was performed according to the standard protocol implemented in the scikit-learn library version 1.4.2 (https://scikit-learn.org/stable/, accessed on 6 March 2026) within Python version 3.11. Global seeds were fixed to ensure reproducibility (random_state = 42, n_jobs = 1).

### 2.7. Machine Learning Models Evaluation

Classification efficiency was evaluated using the following metrics: accuracy, recall, precision, and F1-score. Additionally, the ROC curve was generated and the area under the curve (AUC) was calculated. Classification performance was evaluated using five-fold stratified cross-validation, with grouping by horse. The results are presented as mean ± SD. The same TMJs were not duplicated across datasets. The models were evaluated using the standard formulas implemented in the scikit-learn library version 1.4.2 (https://scikit-learn.org/stable/, accessed on 6 March 2026) within Python version 3.11.

## 3. Results

### 3.1. CT Finding Scores Among Healthy TMJ and TMJ OA Groups

In the healthy TMJ group, no CT findings were observed in 17 TMJs, whereas at least one CT finding was identified in 24 TMJs without histopathological confirmation of OA. Healthy TMJs were bilaterally annotated in 17 horses and unilaterally in 7. In contrast, in the TMJ OA group, all 41 TMJs exhibited at least one CT finding with histopathological confirmation of OA. TMJ OA was bilaterally annotated in 17 horses and unilaterally in 7.

**Figure 3:**
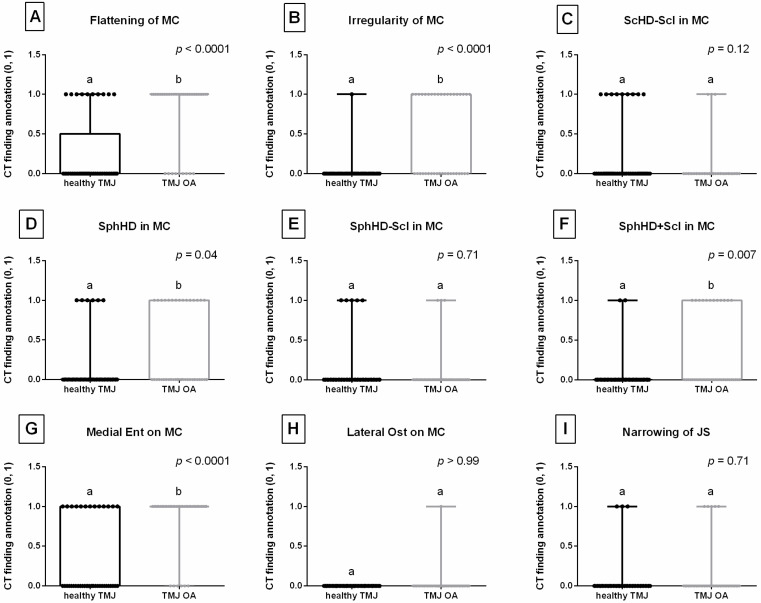
Computed tomography (CT) findings assessed in equine temporomandibular joints (TMJs) representing (**A**) flattening of the mandibular condyle (MC); (**B**) irregularity of MC; (**C**) scattered regions of hypodensity in MC without sclerosis (ScHD − Scl in MC); (**D**) total spherical regions of hypodensity in MC (SphHD in MC); (**E**) spherical regions of hypodensity in MC without sclerosis (SphHD − Scl in MC); (**F**) spherical regions of hypodensity in MC with sclerosis (SphHD + Scl in MC); (**G**) enthesiophytes on the medial aspect of MC (Medial Ent on MC); (**H**) osteophytes on the lateral aspect of MC (Lateral Ost on MC); and (**I**) narrowing of the joint space (JS). Boxes represent median and lower and upper quartiles, while whiskers represent minimum and maximum values. Each point represents one realization. Lowercase letters (a, b) indicate differences between healthy TMJ and TMJ osteoarthritis (OA) groups. Statistical significance was set at *p* < 0.05.

CT finding scores for flattening of MC (Figure 3A), irregularity of MC (Figure 3B), SphHD in MC (Figure 3D), SphHD + Scl in MC (Figure 3F), and Medial Ent on MC (Figure 3G) were higher in the TMJ OA group than in the healthy TMJ group. No differences in CT finding scores between the groups were observed for ScHD − Scl in MC (Figure 3C), SphHD − Scl in MC (Figure 3E), Lateral Ost on MC (Figure 3H), and narrowing of JS (Figure 3I).

### 3.2. Biomarker Levels Among Healthy TMJ and TMJ OA Groups

In the healthy TMJ group, IL-1β, IL-12, and VEGF were below the detection limit in two, three, and two samples, respectively. In contrast, in the TMJ OA group, only VEGF was below the detection limit in 10 samples. All other biomarker levels were within their respective detection ranges.

**Figure 4:**
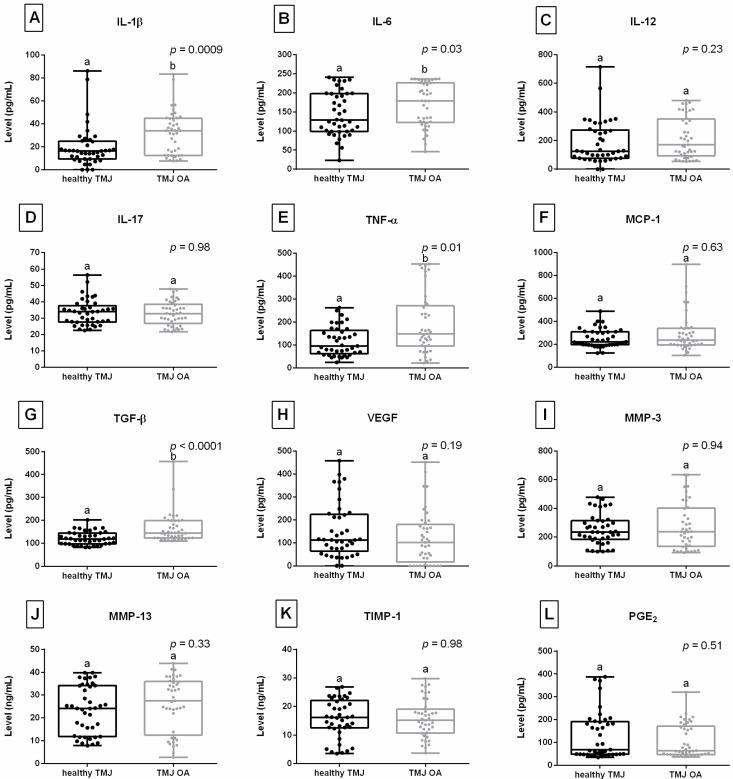
Biomarker levels assessed in synovial fluid from equine temporomandibular joints (TMJs) including (**A**) interleukin 1β (IL-1β); (**B**) interleukin 6 (IL-6); (**C**) interleukin 12 (IL-12); (**D**) interleukin 17 (IL-17); (**E**) tumor necrosis factor α (TNF-α); (**F**) monocyte chemoattractant protein 1 (MCP-1); (**G**) transforming growth factor β1 (TGF-β); (**H**) vascular endothelial growth factor (VEGF); (**I**) matrix metalloproteinase 3 (MMP-3); (**J**) matrix metalloproteinase 13 (MMP-13); (**K**) tissue inhibitor of metalloproteinase 1 (TIMP-1); (**L**) prostaglandin E_2_ (PGE_2_). Boxes represent median and lower and upper quartiles, while whiskers represent minimum and maximum values. Each point represents one realization. Lowercase letters (a, b) indicate differences between healthy TMJ and TMJ osteoarthritis (OA) groups. Statistical significance was set at *p* < 0.05.

Levels of IL-1β (Figure 4A), IL-6 (Figure 4B), TNF-α (Figure 4E), and TGF-β (Figure 4G) were higher in the TMJ OA group than in the healthy TMJ group. No differences in biomarker levels between the groups were observed for IL-12 (Figure 4C), IL-17 (Figure 4D), MCP-1 (Figure 4F), VEGF (Figure 4H), MMP-3 (Figure 4I), MMP-13 (Figure 4J), TIMP-1 (Figure 4K), and PGE_2_ (Figure 4L).

### 3.3. Correlation Structure Among TMJ OA, Age, Laterality, and Biomarker Levels

Specific biomarker levels demonstrated predominantly positive correlations with TMJ OA, age, and with each other; however, none of the studied variables showed a statistically significant correlation with Laterality, as shown in Table 3.

Moderate positive correlations were observed between TMJ OA and age, and between TMJ OA and TGF-β levels. Moderate positive correlations were also found between IL-6 and IL-12 levels, and between IL-17 and MMP-13 levels. In addition, moderate positive correlations were observed between IL-12 and TNF-α levels and between IL-12 and MMP-13 levels, whereas a weak positive correlation was found between IL-12 and TGF-β levels. Weak positive correlations were observed between TMJ OA and IL-1β, IL-6, and TNF-α levels. Weak positive correlations were also found between age and IL-1β, MCP-1, and TGF-β levels. Additional weak positive correlations were observed between IL-1β and IL-12, TNF-α, TGF-β, and MMP-13 levels, as well as between IL-6 and TNF-α levels. Weak positive correlations were also noted between TNF-α and TGF-β levels and between TNF-α and MMP-13 levels, whereas a weak negative correlation was observed between TNF-α and VEGF levels. A weak positive correlation was found between VEGF and MMP-13 levels, while a weak negative correlation was observed between MMP-13 and TIMP-1 levels. No additional significant correlations were identified.

### 3.4. Mixed-Effects Logistic Regression Model of Equine TMJ OA

Among the biomarker levels, when analyzed alongside age and laterality, age was demonstrated as the only estimator of the probability of TMJ OA, as shown in Table 4. Increasing age was associated with a higher OR of TMJ OA, with an estimated OR of 1.30 and a 95% HDI of 1.08–1.54, indicating a robust positive effect.

This suggests that laterality and biomarker levels were not significantly associated with group assignment—healthy TMJs vs. those affected by OA—in the mixed-effects logistic regression model, indicated by the 95% HDI including 1.0. Although there was a lack of laterality effect on equine TMJ OA probability, the substantial random-effect variance confirmed notable between-horse heterogeneity, underscoring the importance of modeling paired TMJs in horses.

### 3.5. Machine Learning-Based TMJ OA Classification

**Figure 5:**
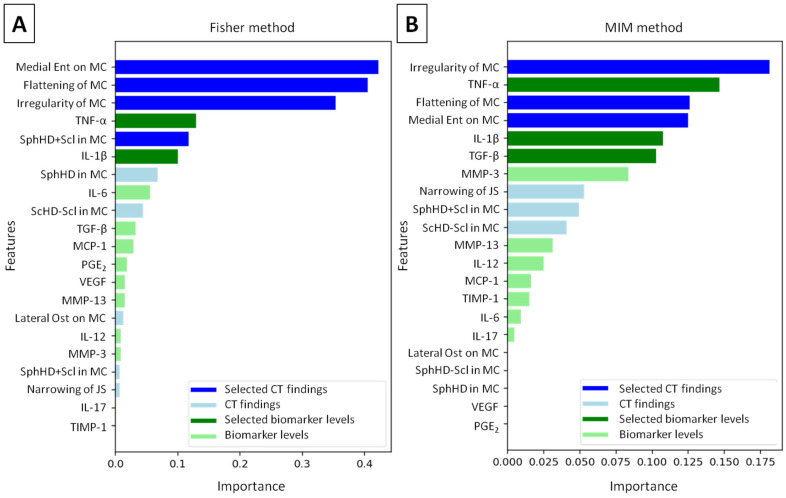
Feature importance assessed using (**A**) Fisher and (**B**) mutual information maximization (MIM) methods. Selected features are highlighted in dark blue and dark green. Footnotes: CT—computed tomography; Ent—enthesiophytes; IL—interleukin; JS—joint space; MC—mandibular condyle; MCP-1—monocyte chemoattractant protein 1; MMP—matrix metalloproteinase; Ost—osteophytes; PGE_2_—prostaglandin E_2_; ScHD—scattered regions of hypodensity; Scl—sclerosis (surrounding hyperdensity); SphHD—spherical regions of hypodensity; TGF-β—transforming growth factor β1; TIMP-1—tissue inhibitor of metalloproteinase 1; TNF-α—tumor necrosis factor α; VEGF—vascular endothelial growth factor; −—without; +—with.

Among the 21 combined radiological (CT findings) and synovial fluid (biomarker level) features, Medial Ent on MC, flattening of MC, irregularity of MC, and SphHD + Scl among CT findings, as well as the levels of TNF-α and IL-1β among the biomarkers, were selected using the Fisher method (Figure 5A). In contrast, irregularity of MC, flattening of MC, and Medial Ent on MC among CT findings, as well as the level of TNF-α, IL-1β, and TGF-β among the biomarkers, were selected using the MIM method (Figure 5B).

Each machine learning model provided TMJ OA classification with accuracy ≥ 0.66, recall ≥ 0.68, precision ≥ 0.67, and F1-score ≥ 0.64. The best accuracy of 0.82 was achieved with the SVM algorithm following feature selection using the MIM method, as shown in Table 5.

**Figure 6:**
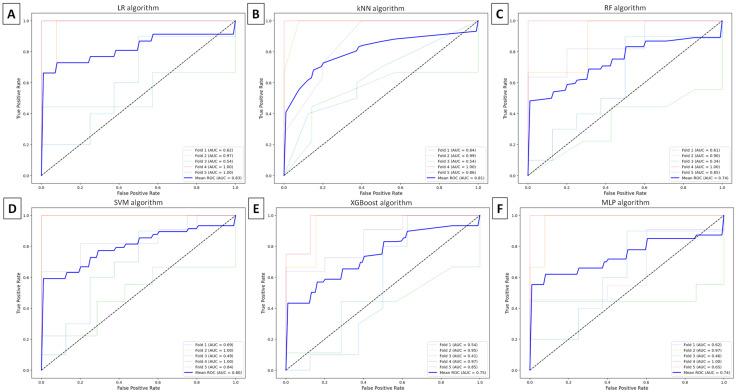
Receiver operating characteristic (ROC) curve, with the area under the curve (AUC), illustrates the five-fold cross-validation of classification models using the Fisher method for feature selection and (**A**) logistic regression (LR), (**B**) k-nearest neighbors (kNNs), (**C**) random forest (RF), (**D**) support vector machines (SVMs), (**E**) extreme gradient boosting (XGBoost), or (**F**) multilayer perceptron (MLP) algorithms for temporomandibular joint (TMJ) osteoarthritis (OA) classification.

**Figure 7:**
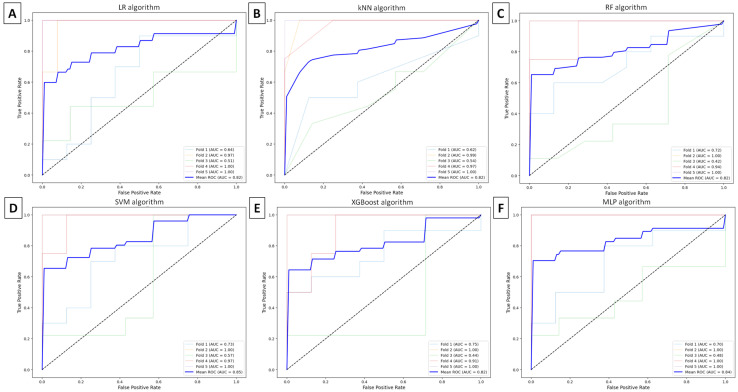
Receiver operating characteristic (ROC) curve, with the area under the curve (AUC), illustrates the five-fold cross-validation of classification models using the mutual information maximization (MIM) method for feature selection and (**A**) logistic regression (LR), (**B**) k-nearest neighbors (kNNs), (**C**) random forest (RF), (**D**) support vector machines (SVMs), (**E**) extreme gradient boosting (XGBoost), or (**F**) multilayer perceptron (MLP) algorithms for temporomandibular joint (TMJ) osteoarthritis (OA) classification.

Based on the ROC curves generated for each machine learning model, AUCs were ≥0.74 when the Fisher method was used (Figure 6) and ≥0.82 when the MIM method was applied (Figure 7). The highest AUC (≥0.85) was achieved by the SVM algorithms following feature selection with the MIM method.

## 4. Discussion

Considering the biological insight of the presented study, it may be observed that synovial fluid levels of IL-1β, IL-6, TNF-α, and TGF-β were higher in the equine TMJs affected by OA than in healthy joints. These results are consistent with the human studies demonstrating increased levels of IL-1β [33,34,35,36], IL-6 [33,34,35,37], TNF-α [33,34,35,36], and TGF-β [39,40] in synovial fluid collected during the course of TMJ OA. However, the obtained results cannot be compared directly with the previous equine studies, as there is a substantial gap in biomarker research in equine TMJ OA [1,50].

A single study on healthy equine TMJs reported undetectable levels of IL-6 and TNF-α in 15 and 11 samples, respectively, and found no differences in IL-1, IL-6, IL-8, TNF-α, and TGF-β levels between horses aged 2–3 years and those aged 9–21 years [24]. In the present study, undetectable levels of IL-1β and IL-12 were observed in two and three samples, respectively, all of which were annotated as healthy. Notably, undetectable levels of any biomarker were not found in samples annotated as TMJ OA, which is in line with the previous human study [53]. Undetectable levels of IL-1β, IL-2, IL-6, IL-10, and interferon γ (IFN-γ) have been reported in some synovial fluid samples from healthy human TMJs, indicating that these cytokines are not generally produced during normal homeostasis [53]. Consequently, the production of these cytokines, particularly IL-1β [33,34,35,36] and IL-6 [33,34,35,37], has been suggested to be specific to human TMJ OA, similar to TNF-α [33,34,35,36] and TGF-β [39,40]. TNF-α [53] and TGF-β [39,42], however, are also present in healthy joints, although at lower levels.

In this study, a moderate positive correlation was observed between TMJ OA and TGF-β level, while weak positive correlations were found between TMJ OA and IL-1β, IL-6, and TNF-α levels. A previous equine study examining the effects of lipopolysaccharide-induced TMJ inflammation on IL-6, TNF-α, and TGF-β levels demonstrated that increased TNF-α and TGF-β levels reflect the activation and recruitment of specific inflammatory cells characteristic of acute inflammation [49]. In contrast, the chronic inflammatory process observed in OA in the present study was characterized by increased levels of IL-1β, IL-6, TNF-α, and TGF-β. However, the specific relationship between these biomarkers and inflammatory cell infiltration was not investigated in the present study. Therefore, further research should explore inflammatory patterns in more biologically oriented study designs, for example, in relation to specific histopathological findings and the type and intensity of immune cell infiltration.

Moreover, previous human studies have demonstrated correlations between biomarker levels—such as IL-1β, IL-6, and TNF-α—and radiographic severity of OA [54]. However, in equine studies, including the present one, correlations between biomarker levels and CT findings have not yet been evaluated. Nevertheless, in the present study, specific biomarker levels demonstrated predominantly positive correlations with each other, e.g., positive correlations between IL-1β and IL-12/TNF-α/TGF-β levels; IL-6 and TNF-α/IL-12 levels; TNF-α and TGF-β/IL-12 levels; and TGF-β and IL-12 levels. This suggests a network of interconnections between the studied biomarkers, which requires further in-depth investigation. In human TMJ OA, the inflammatory response is thought to involve complement activation and macrophages. Activated macrophages produce cytokines, including the pro-inflammatory IL-1β, IL-6, and TNF-α [54], mediated through TGF-β [39,42,48,50] and nuclear factor kappa B (NF-κB) [38,50,54] signaling pathways. Interestingly, microRNAs (miRNAs), including miR-146a, have been shown to be upregulated in equine OA and may affect inflammatory responses by targeting the NF-κB signaling pathway, thereby influencing the production of IL-6 and TNF-α [55]. However, this study was conducted on the equine middle carpal joint [55], which cannot be directly translated to the equine TMJ OA model.

Importantly, a recent equine study demonstrated that TNF-α and TGF-β levels in TMJ synovial fluid differ from those observed in other joints of the appendicular skeleton, suggesting a distinct course of acute inflammation in these joint types. The authors proposed that these differences may result from the unique characteristic of the TMJ as a non-weight bearing and fibrocartilage-surfaced joint. Therefore, findings from equine weight-bearing, hyaline cartilage-surfaced appendicular joints should be translated to TMJ research with caution [49]. This caution is particularly important in light of the growing number of metabolomic, proteomic, and miRNA sequencing studies investigating synovial fluid in OA-affected horses [53,55]. Protein [53] and miRNA [55] profiles in equine OA models have already been established in joints of the appendicular skeleton—specifically in the metacarpophalangeal joint [53] and the middle carpal joint [55]. In humans, proteomics analyses of synovial fluid from appendicular joints have enabled differentiation between healthy joints and those affected by OA, even when inflammation is present in both [56,57], as well as distinction between OA and inflammatory rheumatoid arthritis [58]. Therefore, comparable omics-based studies focused specifically on the equine TMJ are warranted, as synovial fluid proteomics may offer clear potential for achieving more specific diagnostic insights.

Considering risk estimation of equine TMJ OA, the present study demonstrated that age was the only predictor of disease probability. This finding is consistent with human TMJ OA studies in which age has been considered a predisposing factor in disease modeling [31,32]. However, in a previous equine TMJ study, the only age-related differences reported concerned synovial fluid IL-8 levels [24]. These differences were observed only between foals and older horses and were not detected between adult horse groups [24], which aligns with human studies showing no direct age-related effect on cytokine levels in TMJ synovial fluid [33]. Although no age-based grouping was applied in the present study, a moderate positive correlation was observed between age and TMJ OA, while weak positive correlations were also found between age and IL-1β, TGF-β, and MCP-1 levels. These findings suggest that future studies on equine TMJ OA could benefit from transforming descriptive demographic variables, such as age, into computational numerical models, similar to approaches used in human machine learning studies [31,32].

The results of the present study also underscore the importance of modeling paired TMJs in horses, as both joints originate from the same individual. Although laterality showed no effect on the probability of equine TMJ OA, notable between-horse heterogeneity indicated that the observations were not fully independent. This heterogeneity was confirmed by the substantial random-effect variance observed in the GLMM, which appropriately accounted for within-horse associations. The GLMM approach was able to capture more complex interactions within the dataset that were not identified through correlation analyses alone. In the present study, none of the analyzed variables showed a statistically significant correlation with laterality. Given that laterality was not significantly associated with either healthy TMJs or TMJ OA classification, the probability of TMJ OA appears to be independent of whether the right or left joint is affected. However, the potential influence of bilateral disease occurrence was not specifically investigated. In this dataset, healthy TMJs and those affected by OA were annotated bilaterally in 17 horses and unilaterally in 7 horses. A similar balance was observed between healthy TMJs and those affected by OA, all collected from 41 horses. Although these distributions were not determined by the study’s selection criteria, they cannot be considered representative of the broader equine population because the specimens were collected from a commercial slaughterhouse, where predominantly older horses or horses no longer suitable for work are typically submitted. In the general equine population—including breeding, pleasure, racing, and sport horses—the prevalence of TMJ OA is expected to be substantially lower, as TMDs are rarely reported in horses [1].

Although none of the studied biomarkers was included in the probabilistic model of TMJ OA, incorporation of synovial fluid levels of IL-1β, TNF-α, and TGF-β into a computer-aided diagnostic protocol may enable diagnostic performance that exceeds that of conventional diagnostic methods. In humans, the clinical diagnosis of TMJ OA without imaging has been reported to achieve a sensitivity of 0.55 and a specificity of 0.61 [59], whereas comparable data have not yet been published for horses [1]. Incorporating CT findings into the clinical diagnosis of human TMJ OA increases diagnostic sensitivity to 0.67–0.90 and specificity to 0.73–0.93 when CT and clinical findings are combined and assessed against histopathological confirmation of OA [60]. Our preliminary study in horses demonstrated that, when all CT findings are considered, CT-based diagnosis of TMJ OA achieved a sensitivity of 0.79 and a specificity of 0.50 relative to histopathologically confirmed TMJ OA [51]. Further incorporation of synovial fluid biomarkers into the clinical diagnosis of human TMJ OA has enabled predictive models, achieving an accuracy of 0.76 and an AUC of 0.83 in one study [32] and an accuracy of 0.82 and an AUC of 0.87 in another [31], both for predicting TMJ OA status. These values were considered indicative of strong diagnostic performance [31,32]. In the present study, the best-performing model achieved an accuracy of 0.82 and an AUC of 0.85. According to the performance criteria applied by Bianchi et al. [31] and Al Turkestani et al. [32], these results can be considered indicative of strong diagnostic performance and exceed the diagnostic capability of conventional methods based solely on CT findings [51]. However, the implementation of deep learning models—a more advanced and promising subset of machine learning—has achieved CT-based prediction accuracy of up to 0.95 for TMJ OA in a human study [61], suggesting a promising direction for further research in equine TMJ OA as well. Notably, in previous human studies [31,32], as well as in the present equine study, machine learning models have successfully transformed CT findings and biomarker levels from descriptive measurements into a computer-aided diagnostic tool capable of predicting TMJ OA.

In the TMJs analyzed in this study, healthy TMJs differed from joints with histopathologically confirmed OA in five CT findings—specifically, flattening of MC, irregularity of MC, presence of subchondral bone cysts (particularly those with surrounding sclerosis), and presence of medial enthesiophytes—as well as in the levels of four synovial fluid biomarkers, namely IL-1β, IL-6, TNF-α, and TGF-β. Importantly, this group of nine combined CT findings and biomarker levels overlapped with the features that achieved the highest importance using both feature selection methods employed in this study. Based on the results of the Fisher method, a univariate feature selection method [62], flattening and irregularity of MC, presence of subchondral bone cysts and medial enthesiophytes, as well as levels of IL-1β and TNF-α, were incorporated into the machine learning models. In contrast, according to the results of the MIM method, a multivariate feature selection method [62], flattening and irregularity of MC, presence of medial enthesiophytes, as well as levels of IL-1β, TNF-α, and TGF-β were included in the models. Univariate methods are simple and computationally efficient; however, because they evaluate each feature independently, they focus solely on feature significance and cannot eliminate redundancy among features or detect complex feature interactions, which are addressed by multivariate methods [62]. This may explain why the more advanced MIM method outperformed the simpler Fischer method when applied to the combined dataset of CT findings and biomarker levels. At this stage of research, it is possible that the Fisher method’s inability to account for feature interactions resulted in the loss of important information in the analyzed dataset [62], a limitation that was not present using the MIM method. Consequently, the MIM method was able to detect more complex interactions between CT findings and biomarker levels in equine TMJs. These interactions may reflect underlying mechanistic interactions capturing early inflammatory, structural, and neurovascular changes—e.g., the radiomics-cartilage degradation relationship—previously demonstrated in human TMJs [32] and successfully applied in computer-aided diagnosis of human TMJ OA [31,32]. However, further research is required to investigate variable feature inclusion thresholds and dimensionality reduction techniques. Such techniques, including principal component analysis (PCA) and linear discriminant analysis (LDA), consider a limited number of eigenvectors and may improve feature selection outcomes [63].

Although CT is currently considered the reference standard for TMJ imaging in both human [59] and equine [1] clinical practice for diagnosing OA, the clinical relevance of some CT findings in horses remains unclear [16]. In humans, a healthy TMJ is generally defined as an asymptomatic joint without radiographic evidence of OA [29]. In contrast, certain CT findings—referred to as ‘CT anatomical variations’—were identified in at least one TMJ in 40% of asymptomatic horses, accounting for 29% of the 2036 imaged joints [16]. Among these ‘CT anatomical variations’, flattening of MC and the presence of subchondral bone cysts and medial enthesiophytes have been reported. Notably, in the present study, these CT findings successfully passed the feature selection process and were included in the classification models. Therefore, further research should be pursued in two related directions. First, it may be justified to exclude certain ‘CT anatomical variations’ from the dataset to avoid misclassifying age-related radiological features of TMJ remodeling [16] as predictors of OA. Second, the clinical relevance of each CT finding should be established, for example, through the use of intra-articular analgesia [15,16,23,26], particularly because some horses classified as asymptomatic may in fact have subclinical TMJ OA [16]. Among the 1018 horses included in the referenced study, some may have experienced mild TMJ pain without exhibiting moderate or severe clinical signs of TMJ pain or masticatory dysfunction, especially considering that 10.7% of these horses were imaged for head-shaking syndrome [16].

In such equivocal cases, assessment of biomarkers of symptomatic TMJ OA could be valuable for distinguishing clinically relevant CT findings from ‘CT anatomical variations’ identified in asymptomatic horses. This approach is particularly feasible because synovial fluid collection does not require a separate arthrocentesis and can be performed during the same procedure as intra-articular analgesia [15,19,26] or intra-articular medication [15,17]. A biomarker of particular clinical relevance is synovial fluid PGE_2_, which has been shown to increase in humans with chronic TMJ OA associated with TMJ pain and masticatory dysfunction [45]. Although neither the present study nor a previous equine study [22] demonstrated an effect of TMJ OA categorization or a one-hour mechanical overload [22] on equine TMJ PGE_2_ levels, the role of PGE_2_ as a biomarker of symptomatic TMJ OA warrants further in vivo investigation. This is especially important given that all horses in the previous in vivo study were considered asymptomatic [22]. Moreover, in the present cadaver study, potential clinical signs of equine TMD—such as joint swelling and mild to moderate TMJ dysfunctions [1], including head-shaking syndrome [17,21], problems with bit contact [15,16], riding-related behavior [16,17], or mastication [13,14,16,17]—could not be evaluated. Therefore, further equine studies should be expanded by integrating clinical data, broadening the biomarker panel to include omics approaches, and incorporating radiomic features derived from CT findings. It would also be advisable to broaden the range of feature selection methods, for example, by incorporating PCA and LDA [63], as well as to evaluate additional machine learning algorithms, including deep learning models [61]. However, the successful integration of these approaches within a high-quality study design will necessarily require extensive in vivo equine studies.

## 5. Conclusions

Synovial fluid levels of IL-1β, IL-6, TNF-α, and TGF-β increase during the course of equine TMJ OA, making these proteins potential biomarkers of the disease. Among them, IL-1β and TGF-β showed positive correlations with TMJ OA, age, and with each other; however, only age was recognized as a predictor of equine TMJ OA probability. Although biomarker levels were not included in the probabilistic model, incorporating synovial fluid levels of IL-1β, TNF-α, and TGF-β into a computer-aided diagnostic protocol enabled diagnostic performance exceeding that of conventional diagnostic methods based solely on CT findings. Therefore, the proposed classification model may be considered a useful tool for supporting the diagnosis of equine TMJ OA. However, due to substantial gaps in equine research, further studies are required before this promising diagnostic approach can be implemented in clinical practice. In particular, future research should focus on defining disease-specific biomarker profiles, further optimizing machine learning models, and confirming the clinical relevance of individual CT findings. Despite potential limitations, the best-performing machine learning model used in this study successfully transformed descriptive CT findings and biomarker levels into a computer-aided diagnostic approach applicable to equine TMJ OA classification.

## Figures and Tables

**Table 1:** Computed tomography (CT) findings assessed in equine temporomandibular joints (TMJs).

CT Findings	Descriptor	Annotation
Flattening of MC	Flattening of the mandibular condyle	0; 1
Irregularity of MC	Irregularity of the mandibular condyle	0; 1
ScHD − Scl in MC	Scattered regions of hypodensity in the mandibular condyle without sclerosis	0; 1
SphHD in MC	Spherical regions of hypodensity in the mandibular condyle regardless of sclerosis	0; 1
SphHD − Scl in MC	Spherical regions of hypodensity in the mandibular condyle without sclerosis	0; 1
SphHD + Scl in MC	Spherical regions of hypodensity in the mandibular condyle with sclerosis	0; 1
Medial Ent on MC	Enthesiophytes on the medial aspect of the mandibular condyle	0; 1
Lateral Ost on MC	Osteophytes on the lateral aspect of the mandibular condyle	0; 1
Narrowing of JS	Narrowing of the joint space	0; 1

Footnotes: Ent—enthesiophytes; JS—joint space; MC—mandibular condyle; Ost—osteophytes; ScHD—scattered regions of hypodensity; Scl—sclerosis (surrounding hyperdensity); SphHD—spherical region of hypodensity; −—without; +—with; 0—absent; 1—present.

**Table 2:** Enzyme-linked immunosorbent assay (ELISA) kits used for determining selected biomarker levels in synovial fluid collected from equine temporomandibular joints (TMJ).

Biomarkers	Catalog Number	Sensitivity	Detection Range	Intra-Assay CV	Inter-Assay CV
IL-1β	ELK6249	0.56 pg/mL	1.57–100 pg/mL	<8%	<10%
IL-6	ELK5926	3 pg/mL	7.82–500 pg/mL	<8%	<10%
IL-12	ELK5876	13.3 pg/mL	31.25–2000 pg/mL	<8%	<10%
IL-17	ELK5888	5.8 pg/mL	15.63–1000 pg/mL	<8%	<10%
TNF-α	ELK5856	2.6 pg/mL	7.82–500 pg/mL	<8%	<10%
MCP-1	ELK0634	13.4 pg/mL	31.25–2000 pg/mL	<8%	<10%
TGF-β	ELK5837	28 pg/mL	78.13–5000 pg/mL	<8%	<10%
VEGF	ELK5807	5.4 pg/mL	15.63–1000 pg/mL	<8%	<10%
MMP-3	ELK5809	12.9 pg/mL	31.25–2000 pg/mL	<8%	<10%
MMP-13	ELK5943	0.29 ng/mL	0.79–50 ng/mL	<8%	<10%
TIMP-1	ELK10754	1.31 ng/mL	3.13–200 ng/mL	<8%	<10%
PGE_2_	ELK7886	10.8 pg/mL	31.25–2000 pg/mL	<8%	<10%

Footnotes: CV—coefficient of variation; IL—interleukin; MCP-1—monocyte chemoattractant protein 1; MMP—matrix metalloproteinase; PGE_2_—prostaglandin E_2_; TGF-β—transforming growth factor β1; TIMP-1—tissue inhibitor of metalloproteinase 1; TNF-α—tumor necrosis factor α; VEGF—vascular endothelial growth factor.

**Table 3:** Spearman correlation coefficients (ρ) for associations between temporomandibular joint osteoarthritis (TMJ OA) annotations, horses’ age, sample laterality, and biomarker levels. The record was highlighted by a bold font when *p* < 0.05.

(ρ; *p*)	TMJ OA	Age	Laterality	IL-1β	IL-6	IL-12	IL-17	TNF-α	MCP-1	TGF-β	VEGF	MMP-3	MMP-13	TIMP-1	PGE_2_
TMJ OA	X	**0.60; <0.01**	0.20; 0.83	**0.36; <0.01**	**0.24; 0.03**	0.13; 0.23	−0.02; 0.89	**0.27; 0.01**	0.05; 0.63	**0.46; <0.01**	−0.15; 0.19	0.01; 0.94	0.11; 0.33	−0.02; 0.89	−0.07; 0.50
Age		X	0; 1	**0.30; 0.01**	0.14; 0.21	−0.01; 0.91	−0.03; 0.78	0.18; 0.10	**0.23; 0.03**	**0.27; 0.02**	−0.06; 0.59	−0.08; 0.50	0.15; 0.17	−0.06; 0.61	−0.15; 0.18
Laterality			X	−0.04; 0.70	−0.04; 0.70	−0.07; 0.52	−0.07; 0.54	0; 0.99	0.05; 0.68	−0.05; 0.65	0.10; 0.36	0.04; 0.74	−0.04; 0.75	0; 0.97	0.04; 0.72
Cytokines															
IL-1β				X	0.19; 0.10	**0.28; 0.01**	0.02; 0.83	**0.22; 0.04**	0.19; 0.08	**0.26; 0.02**	0.12; 0.27	−0.10; 0.39	**0.23; 0.03**	0.01; 0.96	−0.14; 0.20
IL-6					X	**0.48; <0.01**	−0.07; 0.55	**0.38; <0.01**	0.11; 0.33	0.18; 0.11	−0.10; 0.35	−0.21; 0.05	0.18; 0.10	0.08; 0.46	0.02; 0.86
IL-12						X	0.19; 0.08	**0.52; <0.01**	0.12; 0.30	**0.25; 0.02**	0.10; 0.37	−0.15; 0.19	**0.52; <0.01**	0.20; 0.08	−0.15; 0.17
IL-17							X	0.08; 0.48	−0.21; 0.06	−0.02; 0.88	0; 0.99	0.09; 0.40	**0.41; <0.01**	0.21; 0.06	−0.21; 0.06
TNF-α								X	0.20; 0.07	**0.24; 0.03**	**−0.23; 0.04**	−0.04; 0.72	**0.36; <0.01**	−0.04; 0.73	−0.03; 0.77
Chemokines															
MCP-1									X	0.13; 0.25	−0.02; 0.86	0.13; 0.24	0.12; 0.28	−0.20; 0.07	−0.11; 0.31
Growing factors															
TGF-β										X	−0.08; 0.46	−0.20; 0.07	0.14; 0.21	−0.04; 0.69	−0.14; 0.21
VEGF											X	0.04; 0.70	**0.24; 0.03**	0.12; 0.29	0.01; 0.92
Extracellular matrix remodeling enzymes															
MMP-3												X	−0.05; 0.81	−0.14; 0.05	−0.20; 0.67
MMP-13													X	**−0.03; 0.04**	−0.22; 0.22
TIMP-1														X	0.22; 0.07
Other mediators															
PGE_2_															X

Footnotes: Biomarker levels include interleukin 1β (IL-1β), interleukin 6 (IL-6), interleukin 12 (IL-12), interleukin 17 (IL-17), tumor necrosis factor α (TNF-α), monocyte chemoattractant protein 1 (MCP-1), transforming growth factor β1 (TGF-β), vascular endothelial growth factor (VEGF), matrix metalloproteinase 3 (MMP-3), matrix metalloproteinase 13 (MMP-13), tissue inhibitor of metalloproteinase 1 (TIMP-1), and prostaglandin E_2_ (PGE2).

**Table 4:** Odds ratios (ORs) and 95% highest density intervals (95% HDIs) for associations between temporomandibular joint osteoarthritis (TMJ OA) annotations, horses’ age, sample laterality, and biomarker levels. The record was highlighted by a bold font when the credibility was true.

	OR	(95% HDI)	Credibility
Age	**1.30**	**(1.09–1.55)**	**true**
Laterality	1.31	(0.20–7.97)	false
Cytokines			
IL-1β	1.72	(0.52–5.57)	false
IL-6	2.04	(0.63–6.67)	false
IL-12	0.98	(0.25–3.63)	false
IL-17	1.10	(0.31–3.61)	false
TNF-α	1.78	(0.42–6.58)	false
Chemokines			
MCP-1	1.87	(0.59–6.68)	false
Growing factors			
TGF-β	0.63	(0.23–1.57)	false
VEGF	0.97	(0.29–3.38)	false
Extracellular matrix remodeling enzymes			
MMP-3	1.87	(0.52–7.31)	false
MMP-13	0.85	(0.22–3.01)	false
TIMP-1	0.94	(0.29–3.43)	false
Other mediators			
PGE_2_	0.66	(0.19–2.20)	false

Footnotes: Biomarker levels include interleukin 1β (IL-1β), interleukin 6 (IL-6), interleukin 12 (IL-12), interleukin 17 (IL-17), tumor necrosis factor α (TNF-α), monocyte chemoattractant protein 1 (MCP-1), transforming growth factor β1 (TGF-β), vascular endothelial growth factor (VEGF), matrix metalloproteinase 3 (MMP-3), matrix metalloproteinase 13 (MMP-13), tissue inhibitor of metalloproteinase 1 (TIMP-1), and prostaglandin E_2_ (PGE_2_).

**Table 5:** Classification metrics (mean ± SD) of equine temporomandibular joints (TMJs) osteoarthritis (OA) based on a combination of CT findings and biomarker levels. The best accuracy for each classifier is highlighted in bold. The best accuracy across classifiers is highlighted with underlining.

Classifier	Feature Selection	Accuracy	Recall	Precision	F1-Score
LR	Fisher	**0.80 ± 0.16**	0.81 ± 0.17	0.79 ± 0.15	0.78 ± 0.16
	MIM	0.77 ± 0.15	0.78 ± 0.17	0.75 ± 0.15	0.76 ± 0.15
kNN	Fisher	**0.78 ± 0.16**	0.80 ± 0.16	0.78 ± 0.14	0.77 ± 0.16
	MIM	**0.78 ± 0.19**	0.79 ± 0.20	0.78 ± 0.19	0.78 ± 0.19
RF	Fisher	**0.72 ± 0.17**	0.74 ± 0.17	0.71 ± 0.17	0.71 ± 0.17
	MIM	0.69 ± 0.22	0.70 ± 0.23	0.68 ± 0.22	0.68 ± 0.22
SVM	Fisher	0.77 ± 0.17	0.76 ± 0.18	0.76 ± 0.16	0.75 ± 0.17
	MIM	**0.82 ± 0.18**	0.82 ± 0.18	0.82 ± 0.18	0.82 ± 0.18
XGBoost	Fisher	0.70 ± 0.12	0.71 ± 0.13	0.69 ± 0.10	0.68 ± 0.10
	MIM	**0.72 ± 0.27**	0.72 ± 0.27	0.72 ± 0.27	0.82 ± 0.18
MLP	Fisher	0.66 ± 0.17	0.68 ± 0.17	0.67 ± 0.17	0.64 ± 0.18
	MIM	**0.76 ± 0.18**	0.77 ± 0.20	0.74 ± 0.18	0.75 ± 0.18

Footnotes: kNN—k-nearest neighbors; LR—logistic regression; MIM—mutual information maximization; MLP—multilayer perceptron; RF—random forest; SVM—support vector machine; XGBoost—extreme gradient boosting.

## Data Availability

The data presented in this study are available on request from the corresponding author.
